# Enfortumab vedotin–related cutaneous toxicity correlates with overall survival in patients with urothelial cancer: a retrospective experience

**DOI:** 10.3389/fonc.2024.1377842

**Published:** 2024-06-12

**Authors:** Evangelia Vlachou, Burles Avner Johnson, David McConkey, Yuezhou Jing, Andres Matoso, Noah M. Hahn, Jean Hoffman-Censits

**Affiliations:** ^1^ Department of Urology, The Johns Hopkins Greenberg Bladder Cancer Institute, Baltimore, MD, United States; ^2^ Department of Oncology, Johns Hopkins University Sidney Kimmel Comprehensive Cancer Center, Baltimore, MD, United States; ^3^ The James Buchanan Brady Urological Institute, Johns Hopkins University, Baltimore, MD, United States; ^4^ Departments of Pathology, Urology and Oncology, Johns Hopkins University School of Medicine, Baltimore, MD, United States

**Keywords:** advanced/metastatic urothelial cancer, bladder cancer, cutaneous adverse events, disease response, enfortumab vedotin (EV), overall survival (OS), progression-free survival (PFS), skin toxicity

## Abstract

**Introduction:**

Enfortumab vedotin (EV) is an antibody drug conjugate approved for advanced urothelial cancer, consisting of a monomethyl auristatin E payload linked to a human monoclonal antibody targeting nectin-4. No validated biomarker predictive of or correlated with response exists for EV. Cutaneous toxicity is among the most common EV-related toxicities and typically emerges in early cycles. This retrospective experience of patients with urothelial cancer treated with EV monotherapy evaluated whether EV-related cutaneous toxicity correlated with improved outcomes including progression-free (PFS) and overall (OS) survival and overall response rate (ORR).

**Patients and methods:**

Patients treated with EV monotherapy at Johns Hopkins were identified, and baseline characteristics, treatment, and toxicity details were extracted through chart review. Univariable Cox hazard ratios (HRs) were calculated for assessing the effect of baseline patient characteristics and cutaneous toxicity in PFS and OS. Based on the univariable analysis and known risk factors, all subsequent analyses were adjusted for: Eastern Cooperative Oncology Group performance status, visceral metastases at baseline, gender as well as EV dose, and weight to account for dosing differences. Multivariable Cox proportional HRs were used for comparing PFS and OS between patients with and without cutaneous toxicity, assessing toxicity and EV dose as a time-dependent variables. Adjusted p-values were calculated to compare ORR and disease control rate (DCR) between groups using the Poisson regression model.

**Results:**

Of the 78 patients analyzed, 42 (53.8%) experienced EV-related cutaneous toxicity that appeared early during treatment (median time to occurrence 0.5 months from EV initiation). Cutaneous toxicity correlated with significantly improved OS [HR, 0.48; 95% confidence interval (CI), 0.25, 0.9; P = 0.0235], ORR (68.3% vs. 20.7%, P = 0.0033) and DCR (82.9% vs. 48.3%, P = 0.0122). Median PFS was numerically longer in the cutaneous toxicity group (6.3 vs. 1.7 months), although no significance was achieved in the multivariable analysis (HR, 0.62; 95% CI: 0.35, 0.108; P = 0.0925).

**Conclusion:**

In this retrospective study, EV-related cutaneous toxicity was associated with improved patient outcomes. Confirming this observation and understanding its mechanism could lead to discovery of a new clinical biomarker of EV response that can emerge in the first cycle.

## 1 Introduction

Locally advanced (T3b, T4, and N1−N3) and metastatic urothelial cancer (la/mUC) of the bladder, ureter, and renal pelvis is an aggressive disease with poor prognosis. Median overall survival (OS) is 13 months with cis- or carboplatin-based chemotherapy that, until recently, had been the first-line treatment for decades (1, 2). Historically, chemotherapy was the only treatment option for la/mUC; however, in the recent years, new treatment options have emerged including checkpoint inhibitors, antibody drug conjugates (ADCs), and targeted therapies. The addition of maintenance therapy with the checkpoint inhibitor avelumab, following four to six cycles of platinum-based chemotherapy in patients who did not experience disease progression, further prolonged median OS to 21.4 months in that group (3). Recently, the addition of nivolumab to standard cisplatin and gemcitabine chemotherapy led to an improvement in median OS of 21.7 months versus 18.7 months with standard chemotherapy alone (4).

Enfortumab vedotin (EV) is an antibody drug conjugate (ADC) approved in Europe and the US for patients with la/mUC, with unprecedented response rates of 44%–52% following platinum-based chemotherapy and/or programmed cell death protein 1 (PD-1) and ligand 1 (PD-L1) inhibitors (1, 2, 5, 6). In early 2023, EV in combination with pembrolizumab (P) was granted accelerated FDA approval as first-line treatment for cisplatin ineligible patients with la/mUC based on phase 1b/II data (7, 8). In December 2023, full FDA approval was granted as first-line treatment for all patients with la/mUC based on the phase 3 EV-302 trial showing that EV in combination with pembrolizumab (P) significantly improved OS and progression-free survival (PFS) compared to first-line platinum chemotherapy (9). EV consists of the chemotherapeutic monomethyl auristatin E (MMAE) linked to a human monoclonal antibody targeting nectin-4, which is expressed on the surface of urothelial cancer cells (10, 11). Single-agent dosing is weight based, 1.25 mg/kg intravenously on days 1, 8, and 15 of a 4-week cycle.

Despite nectin-4 being the target antigen for EV, expression, which is generally high in most urothelial cancers, does not guarantee response (1, 2, 8). Thus, to date, no validated biomarker predicting EV response exists. Given the variety of currently available la/mUC treatment options, such biomarkers would help us identify those patients not destined to respond to EV and who benefit the most from alternative treatment options.

Cutaneous toxicity is a common treatment-related adverse event (TRAE) associated with EV (1, 2, 5, 6). It appears early, mainly within the first or second cycle (12). EV-related cutaneous toxicity can vary in severity and presentation, including maculopapular rash, blisters, dry skin, hyperpigmentation, scaly papules, and rarely life-threatening manifestations like Stevens-Johnson Syndrome and Toxic Epidermal Necrolysis (13). Management of low-grade toxicity is supportive with topical corticosteroids and antihistamines. Grade ≥ 3 events require treatment interruption, oral corticosteroids, and dermatologic consultation. Treatment can resume with dose reduction for grade 3 events that have improved to grade ≤ 1 (13). Peripheral neuropathy is another common TRAE associated with EV, but, unlike cutaneous toxicity, it tends to occur in later cycles. In a recent update of the phase III trial comparing enfortumab to chemotherapy in patients with urothelial cancer (UC) progression post platinum and PD-1 and PD-L1 inhibitors, median time to skin toxicity was 0.43 months, and median time to peripheral neuropathy was 2.81 months (12).

We previously reported a statistically significant ORR increase associated with presence of cutaneous toxicity in a retrospective cohort of 51 patients treated with EV monotherapy (14). We hypothesized that this clinical observation would also translate into a survival signal in those with cutaneous toxicity. Here, we present an expanded retrospective cohort with longer follow up to evaluate whether EV-related cutaneous events correlate with longer PFS and OS. Finally, we also investigated whether a similar association exists between EV-related peripheral neuropathy and outcomes.

## 2 Patients and methods

### 2.1 Patient cohort and data collection

With Institutional Review Board approval, patients with urothelial cancer treated with EV at Johns Hopkins Hospital were identified through the pharmacy database. Baseline patient characteristics, line of therapy, TRAEs, dosing modifications, follow-up dates, and radiographic response were collected through chart review. Study data were collected and managed using REDCap electronic data capture tools hosted at Johns Hopkins.

EV-related cutaneous toxicity was defined as any grade of new or exacerbated dermatologic events following EV cycle 1, day 1 (C1D1), including pruritus, rash, and hyperpigmentation not attributable to another cause. Given that neuropathy is also a very common toxicity, we evaluated it as a comparator toxicity. EV-related neuropathy was defined as new or exacerbated neuropathic symptoms including numbness, tingling, pain, or weakness in the hands or feet following EV C1D1 not attributable to another cause. Neuropathy and cutaneous toxicity were graded according to the Common Terminology Criteria for Adverse Events v5.0. Skin lesions covering <10%, 10–30%, and >30% of body surface area were classified as grades 1, 2, and 3 accordingly. Life-threatening conditions were categorized as grade 4 events. Peripheral neuropathy was defined as grade 1 when asymptomatic; grade 2 in the presence of moderate symptoms, limiting some activities of daily living; and grade 3 when severe symptoms are present or symptoms limiting self-care. Life-threatening events were categorized as grade 4. Radiographic response was assessed by physician-assessed Response Evaluation Criteria in Solid Tumors criteria.

### 2.2 Statistical analysis

Descriptive statistics were used for summarizing baseline patient characteristics for patients with and without EV-related cutaneous toxicity. Univariable Cox proportional hazard model analysis was used to assess the effect of baseline patient characteristics, EV-related cutaneous toxicity, and EV-related peripheral neuropathy on PFS and OS. Kaplan–Meier curves were plotted, and PFS and OS were compared between patients with and without cutaneous toxicity using the log-rank test. PFS was defined as time in months from EV initiation until radiographic disease progression or death of any cause. In the PFS analysis, data for patients who did not experience radiographic disease progression or death were censored at the date of last radiographic evaluation. OS was defined as time in months from EV initiation until death of any cause. In the OS analysis, data for patients who were still alive were censored at the date of last contact with their care team.

Multivariable Cox proportional hazard model analysis was used for comparing PFS and OS between patients with and without cutaneous toxicity. PFS and OS were also compared between patients with and without EV-related peripheral neuropathy, another common EV-related toxicity. Cutaneous toxicity and neuropathy were assessed as time-dependent variables to minimize the risk of immortal-time bias (15). EV dose was also assessed as a time-dependent variable to account for dose changes over time.

Based on the results of the univariable analysis and known la/mUC poor prognosis risk factors, all multivariable models were adjusted for the following: Eastern Cooperative Oncology Group performance status (ECOG PS) (1 versus 0, and ≥2 versus 0), presence of visceral metastases at the time of EV initiation, and gender. To account for potential differences in EV dose between patients, as some were dosed reduced at outset and some in response to any toxicity, all analyses were also adjusted for EV dose (standard of 1.25 mg/kg versus dose reduction of 1 mg/kg in any cycle) and weight at baseline.

Adjusted p-values were calculated for assessing ORR and disease control rate (DCR) between patients with and without EV-related cutaneous toxicity using the Poisson regression model. ORR was defined as the percentage of patients who experienced radiographic complete response (CR) or partial response as best response to EV. DCR was defined as the percentage of patients who had radiographic CR or partial response or stable disease as best response to EV.

Statistical analysis was performed using R 4.2.2 and SAS 9.4 (SAS institute, Cary, NC). All tests were two-sided, and statistical significance was set at p-value <0.05.

## 3 Results

### 3.1 Baseline patient characteristics

From December 2017 until April 2023, 91 patients treated with EV were identified. Patients treated with combination of EV with P (10), intravesical EV (1), or lost to follow-up (2) were excluded. The remaining 78 patients were included in the toxicity, and survival analysis (**Supplementary Figure 1**). Scans were not available for 8/78 patients, and these were excluded from the radiographic response analysis.

Mean age for the entire cohort was 70.7 [interquartile range (IQR): 64.0, 77.8]. **Table 1** summarizes baseline patient characteristics.

**Table 1:** Baseline patient characteristics.

	Cutaneous toxicity N = 42	No cutaneous toxicity N = 36
**Age**, Median (IQR)	70.1 (62.8–75.8)	74.4 (65.2–79.7)
**Gender, n (%)**		
Male	34 (81.0)	24 (66.7)
Female	8 (19.0)	12 (33.3)
**Race, n (%)**		
White	32 (76.2)	26 (72.2)
Black or African American	8 (19.0)	4 (11.1)
Asian	0 (0.0)	4 (11.1)
Other	2 (4.8)	2 (5.6)
**Histologic subtypes/Divergent differentiation (any component)^1^**, n (%)	4 (9.5)	8 (22.2)
**Primary tumor location, n (%)**		
Lower tract	24 (57.1)	21 (58.3)
UTUC	14 (33.3)	14 (38.9)
Both	4 (9.5)	1 (2.8)
**Metastasis present at EV initiation, n (%)**		
Lymph nodes only	8 (19.0)	3 (8.3)
Visceral disease	34 (81.0)	33 (91.7)
Liver	17 (40.5)	19 (52.8)
Bone	10 (23.8)	9 (25.0)
**Line of therapy, n (%)**		
2 Line	7 (16.7)	6 (16.7)
3 Line	27 (64.3)	22 (61.1)
≥ 4 Line	8 (19.0)	8 (22.2)
**ECOG PS at EV initiation, n (%)**		
0	20 (47.6)	6 (16.7)
1	17 (40.5)	22 (61.1)
≥ 2	5 (11.9)	8 (22.2)
**Weight at EV initiation (kg)**, Median (IQR)	82.7 (72.7 - 90.2)	71.2 (57.5 - 86.3)
**Estimated GFR at EV initiation**, Median (IQR)^2^	53.0 (41.0 - 64.0)	52.5 (40.5 - 66.0)
**Patients who initiated EV at full dose**, n (%) ^3^	38 (90.5)	26 (72.2)

IQR, interquartile range; GFR, glomerular filtration rate; UTUC, upper tract urothelial carcinoma; ECOG PS, Eastern Cooperative Oncology Group performance status; EV, enfortumab vedotin.

^1^Histologic subtypes (formerly known as variants) and urothelial carcinoma with divergent differentiation were defined according to the 2022 World Health Organization Classification of Tumors of the Urinary System (16). Patients with small-cell carcinoma of the urinary tract were excluded.

^2^CKD-EPI or MDRN Eqn ml/min/1.73 m^2^—one patient was on hemodialysis (eGFR = 6) at EV initiation and was included in the analysis. That patient did not experience cutaneous toxicity.

^3^Full dose of EV is 1.25 mg/kg, capped at 125 mg/dose.

### 3.2 Treatment-related toxicities

Forty-two patients (53.8%) developed cutaneous toxicity of any grade, with seven (9.0%) patients experiencing ≥ grade 3. No grade 5 events were observed. A summary of EV-related cutaneous events (**Supplementary Table 1**), and dose modifications during EV treatment (**Supplementary Table 2**) is provided. Median time to cutaneous toxicity was 0.5 months (IQR: 0.4, 1.1). Twenty-nine (37.2%) patients developed EV-related peripheral neuropathy with no ≥ grade 3 events. Median time to peripheral neuropathy development was 2.7 months (IQR: 1.28, 4.4).

### 3.3 Overall and progression-free survival

**Figure 1:**
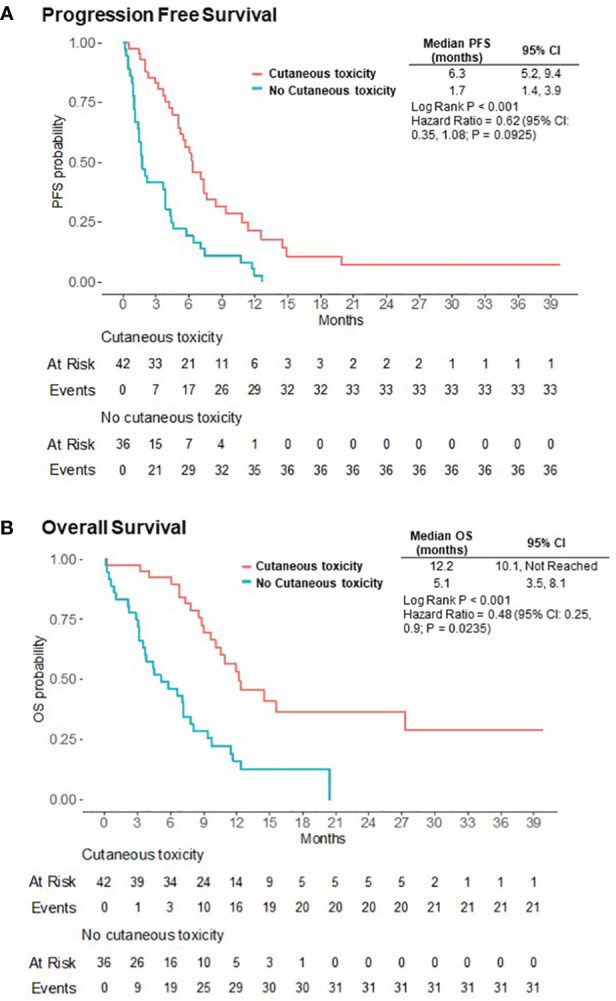
Progression-free (PFS) **(A)** and overall (OS) **(B)** survival for patients who experienced EV-related cutaneous toxicity versus those that did not.

Median PFS [95% confidence interval (95% CI)] was 4.5 months (3.6, 6.2) for the entire cohort, 6.3 months (5.2, 9.4) for patients with cutaneous toxicity, and 1.7 months (1.4, 3.9) without. Median OS (95% CI) was 9.4 months (7.4, 11.9) for the entire cohort, 12.2 months (10.1, not reached) for patients with cutaneous toxicity, and 5.1 months (3.5, 8.1) for patients without (**Figure 1**). The univariable analysis demonstrated PFS and OS benefit associated with the presence of cutaneous toxicity (**Table 2**). Cutaneous toxicity was also associated with a significant increase in OS (HR, 0.48; 95% CI: 0.25, 0.9; P = 0.0235) in the multivariable analysis (**Table 3**). PFS did not remain significant in the multivariable analysis (HR, 0.62; 95% CI: 0.35, 1.08; P = 0.0925). Among the seven patients with ≥ grade 3 cutaneous events, PFS (95% CI) was 7.59 months (5.45, not reached), and OS (95% CI) was 14.5 months (14.5, not reached).

**Table 2:** Univariate Cox proportional hazard model analysis of baseline characteristics and EV-related toxicities for PFS and OS (n = 78).

Characteristics	PFS hazard ratio (95% CI)	p-value	OS hazard ratio (95% CI)	p-value
**Cutaneous toxicity** (time-dependent): yes vs. no	0.46 (0.28, 0.78)	**0.0035**	0.36 (0.2, 0.64)	**0.0006**
**Peripheral neuropathy** (time-dependent): yes vs. no	1.12 (0.63, 2)	0.7061	0.8 (0.43, 1.49)	0.4859
**Race**: White vs. non-white	0.76 (0.43, 1.33)	0.3317	0.81 (0.43, 1.51)	0.5010
**Age** in years, per year	1 (0.98, 1.03)	0.7021	1 (0.97, 1.02)	0.7501
**Gender**: Female vs. male	1.67 (0.97, 2.86)	**0.0633**	1.07 (0.57, 2)	0.8434
**Histologic subtypes/divergent differentiation (any component)**: yes vs. no^1^	1.48 (0.79, 2.79)	0.2211	1.16 (0.56, 2.38)	0.6957
**Primary location:**				
UTUC vs. bladder	1.44 (0.87, 2.38)	0.2204	0.82 (0.45, 1.49)	0.7970
Bladder and UTUC vs. bladder	1.99 (0.7, 5.66)		0.86 (0.26, 2.81)	
**Metastatic disease:** Visceral disease vs. lymph nodes only	2 (0.99, 4.06)	**0.0538**	3.55 (1.27, 9.89)	**0.0156**
Liver metastasis present: yes vs. no	1.2 (0.75, 1.93)	0.4509	1.22 (0.71, 2.11)	0.4761
Bone metastasis present: yes vs. no	1.16 (0.67, 2)	0.5975	1.25 (0.66, 2.36)	0.4914
**ECOG PS:**				
1 vs. 0	1.77 (1, 3.11)	**0.0627**	3.37 (1.64, 6.91)	**0.0024**
≥2 vs. 0	2.23 (1.07, 4.68)		3.61 (1.5, 8.69)	
**Line of therapy:**				
3 vs. 2	0.81 (0.43, 1.51)	0.1551	0.58 (0.29, 1.17)	0.1877
≥4 vs. 2	1.44 (0.68, 3.04)		0.94 (0.42, 2.12)	
**EV dose** (time-dependent) Reduced (1 mg/kg) vs. full (1.25 mg/kg) dose	1.05 (0.64, 1.74)	0.8379	0.75 (0.42, 1.33)	0.3183
**Weight** in kg, per one unit	0.98 (0.96, 0.99)	**0.0041**	0.98 (0.96, 1)	**0.0311**
**Estimated GFR**, per one unit	0.99 (0.98, 1.01)	0.3574	1 (0.98, 1.01)	0.5335

UTUC, upper tract urothelial carcinoma; ECOG PS, Eastern Cooperative Oncology Group performance status; EV, enfortumab vedotin; GFR, glomerular filtration rate.

^1^Histologic subtypes (formerly known as variants) and urothelial carcinoma with divergent differentiation were defined according to the 2022 World Health Organization Classification of Tumors of the Urinary System (16). Patients with small-cell carcinoma of the urinary tract were excluded.

p-values <0.1 appear in bold and represent characteristics included in the multivariable analysis.

**Table 3:** Multivariable Cox proportional hazard model analysis for PFS and OS with relevant baseline characteristics and EV-related cutaneous toxicity (n = 78).

*Risk factors*		*PFS* *HR (95% CI)*	*p-value*	*OS* *HR (95% CI)*	*p-value*
**Cutaneous toxicity** (time-dependent): yes vs. no	0.62 (0.35, 1.08)		0.0925	0.48 (0.25, 0.9)	**0.0235**
**Gender:** female vs. male	1.06 (0.52, 2.17)		0.8747	0.66 (0.28, 1.55)	0.3370
**Metastatic disease:** visceral metastases vs. lymph nodes only	1.77 (0.8, 3.91)		0.1609	3.03 (0.97, 9.43)	0.0558
**ECOG PS:**					
1 vs. 0	1.26 (0.69, 2.33)		0.2316	2.12 (0.99, 4.56)	**0.0213**
≥2 vs. 0	2.06 (0.9, 4.75)			3.76 (1.46, 9.67)	
**Weight**, per 1 kg	0.98 (0.96, 1.004)		0.1066	0.99 (0.96, 1.01)	0.2676
**EV dose** (time-dependent) Reduced (1 mg/kg) vs. full (1.25 mg/kg) dose	0.94 (0.56, 1.57)		0.8111	0.68 (0.38, 1.23)	0.2080

ECOG PS, Eastern Cooperative Oncology Group performance status; EV, enfortumab vedotin.

Values in bold represent statistical significance (p-value <0.05).

No significant difference was found in PFS (HR, 1.32; 95% CI, 0.72, 2.42; p-value = 0.3747) or OS (HR, 1.04; 95% CI: 0.54, 1.99; p-value = 0.9125) when comparing patients with and without EV-related neuropathy in either the univariable or multivariable analysis with neuropathy and EV dose analyzed as a time-dependent variables (**Supplementary Table 3**).

Of note, higher ECOG PS was associated with significantly shorter OS in the multivariable models taking into account other relevant baseline characteristics, EV-related cutaneous toxicity (**Table 3**), and neuropathy (**Supplementary Table 2**).

### 3.4 Radiographic response

In our cohort, ORR was 48.6%, and DCR was 68.6%, similar to published prospective studies. Patients with cutaneous toxicity had improved physician-assessed cancer response rates versus those without (ORR, 68.3% vs. 20.7%; adjusted p-value = 0.0033; and DCR, 82.9% vs. 48.3%; adjusted p-value = 0.0122) (**Table 4**). All patients with CR experienced cutaneous toxicity. Among patients with ≥ grade 3 cutaneous events, six of the seven were evaluable for radiographic response evaluation with ORR of 100% including one (16.7%) with CR and five (83.3%) with PR. One patient refused further radiographic evaluation and was not evaluable.

**Table 4:** Radiographic response in patients with and without EV-related cutaneous toxicity.

Response	Cutaneous toxicity (%) N = 42	No cutaneous toxicity (%) N = 36
**Overall response rate**	28/41 (68.3)	6/29 (20.7)
95% CI, %	51.9, 81.9	8, 39.7
Adjusted p-value	0.0033	
**Disease control rate**	34/41 (82.9)	14/29 (48.3)
95% CI, %	67.9, 92.8	29.4, 67.5
Adjusted p-value	0.0122	
Best physician-assessed radiographic response		
CR	5 (11.9)	0 (0)
PR	23 (54.8)	6 (16.7)
SD	6 (14.3)	8 (22.2)
PD	7 (16.7)	15 (41.7)
N/A	1 (2.4)	7 (19.4)
Number of EV cycles		
Median (IQR)	5 (3.2–7.0)	2 (1.0–4.0)
Adjusted p-value	<0.001	

CR, complete response; PR, partial response; SD, stable disease; PD, progression of disease; EV, enfortumab vedotin; IQR, interquartile range.

## 4 Discussion

In this study, presence of EV-related cutaneous toxicity was associated with significantly improved ORR, DCR, and OS. To our knowledge, this is the first report of a survival association with EV related cutaneous toxicity. Despite correlating with improved PFS in the univariable analysis, this did not remain significant in the multivariable model (when factoring in relevant baseline characteristics and dose adjustments on C1D1 and beyond). This is potentially due to the small cohort numbers.

In this retrospective survival analysis, we evaluated the two most common EV-related toxicities, and, like the previous studies, we found that cutaneous toxicity tends to occur very early while neuropathy tends to occur in later cycles (12, 17). Both in our cohort and in the phase 3 clinical trial of EV monotherapy, median time to cutaneous toxicity occurrence is <1 month, whereas the first radiographic assessment is typically done after the first two to three cycles/2–3 months of treatment (12).

Given that survival was defined as time from EV treatment initiation (t_0_) to event (progression or death) and treatment-related toxicities naturally occur after t_0_, immortal time bias can occur due to misclassification of patients (15). In other words, it is possible that the observed effect is caused by misclassification of patients to the no-toxicity group if they experience progression or death before they had a chance to develop EV-related toxicity. To account for this type of bias, time-dependent Cox models were used for assessing PFS and OS with cutaneous toxicity and peripheral neuropathy analyzed as time-dependent variables. This statistical approach has been proven to minimize the risk of immortal time bias (15).

All analyses were adjusted to potential confounders including known poor prognosis risk factors: ECOG PS (1 versus 0, and ≥2 versus 0), presence of visceral metastases at the time of EV initiation, and gender. Recognizing that dosing differences between groups could be another potential confounder, as patients who received standard dosing could have increased risk of toxicity and improved cancer response compared to those with dose reduction, we adjusted all analyses to dose of EV (full dose versus dose reduction), and weight as EV dosing is weight-based. Dose reduction was 1 mg/kg for all patients who received dose reduction (either at C1D1 or after treatment initiation). In our analysis, dose was assessed as time-dependent variable. Interestingly, in our cohort, dose level was not associated with significant difference in PFS or OS in the univariable or multivariable analysis. This is consistent with data from a recently published study based on a multicenter real-world cohort, where EV dose intensity did not correlate with survival outcomes (18).

In our cohort, seven (9.0%) patients experienced ≥ grade 3 cutaneous toxicity. This small group had a PFS of 7.59 months, OS of 14.5 months, and ORR of 100%. Although these outcomes are numerically higher than the any-grade cutaneous toxicity group, the small numbers preclude a statistical comparison between higher and lower grade cutaneous toxicity.

Many factors may contribute to EV-related dermatologic events, including targeting of nectin-4 expressed in normal skin and MMAE toxicity. Cutaneous toxicity is common with brentuximab vedotin, another ADC with MMAE payload approved for use in lymphoma (19). Dermatologic TRAEs are even more common with EV + P, recently approved as first-line treatment for advanced urothelial cancer (7). Moreover, prior checkpoint inhibitor treatment had been retrospectively correlated with higher grade cutaneous toxicity in EV treated patients with la/mUC (20). In our retrospective cohort, 89.7% of patients had prior checkpoint exposure; thus, altered immunity may have predisposed patients to EV-related skin toxicity.

Significant effort has been made for identifying biomarkers of response to EV and characterizing efficacy in specific patient subgroups; however, no validated biomarker currently exists (21–24). Despite nectin-4 being the target antigen of EV, nectin-4 expression levels are high in most urothelial tumors, and whether they are predictive of response to EV remains controversial. Nectin-4 expression was found to decrease in metastatic sites compared to the corresponding primary tumors in a multicenter cohort of 47 patients (23). However, it did not correlate with response in prospective studies (1, 2, 8).

Although both cutaneous toxicity and neuropathy are common EV-related events, we show that early EV-related cutaneous toxicity can be indicative of response and survival. We feel that neuropathy, which occurs in later cycles, is more likely a cumulative toxicity in those with clinical benefit to EV and, therefore, longer exposure time to the agent. Study limitations include small sample size, physician-assessed radiographic response, and single-center retrospective cohort. With the EV and EV + P use expanding, understanding the mechanisms of EV-related cutaneous toxicity is important. Confirmation of the survival benefit in larger retrospective and prospective studies as well as understanding the mechanism underlying this observation could reveal promising biomarkers of toxicity and response to EV.

## Data availability statement

The raw data supporting the conclusions of this article will be made available by the authors, without undue reservation.

## Ethics statement

The studies involving humans were approved by Johns Hopkins Institutional Review Board. The studies were conducted in accordance with the local legislation and institutional requirements. Written informed consent for participation was not required from the participants or the participants’ legal guardians/next of kin in accordance with the national legislation and institutional requirements.

## Author contributions

EV: Data curation, Formal analysis, Investigation, Project administration, Validation, Visualization, Writing – original draft, Writing – review & editing. BJ: Investigation, Writing – original draft. DM: Investigation, Writing – original draft. YJ: Formal analysis, Writing – original draft, Writing – review & editing. AM: Investigation, Writing – original draft. NH: Investigation, Writing – original draft. JH: Conceptualization, Investigation, Supervision, Writing – original draft, Writing – review & editing.
