# Supplementary figures and images for: Enfortumab vedotin–related cutaneous toxicity correlates with overall survival in patients with urothelial cancer: a retrospective experience
Source: Front Oncol. 2024 Jun 12;14:1377842. doi: 10.3389/fonc.2024.1377842 (PMC11199536; doi:10.3389/fonc.2024.1377842)

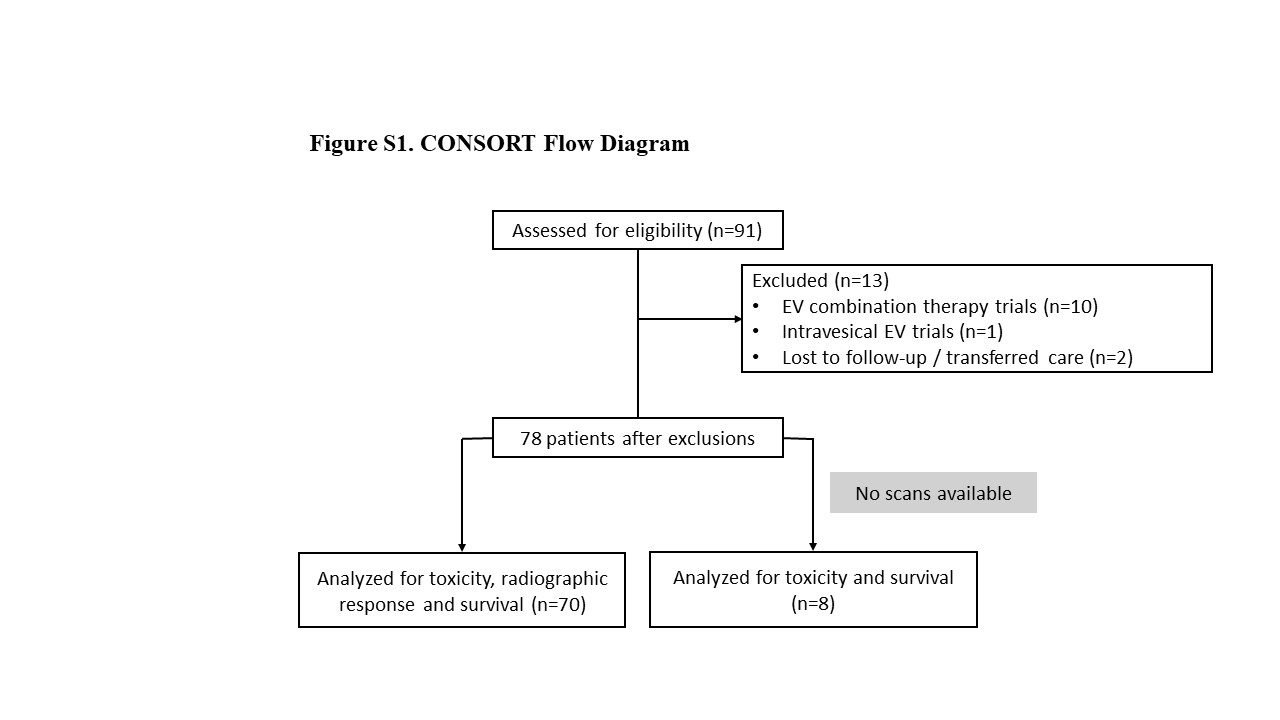

Supplement: Supplementary file 1 [file Image_1.jpeg]
