# Supplementary material for: Enfortumab vedotin–related cutaneous toxicity correlates with overall survival in patients with urothelial cancer: a retrospective experience
Source: Front Oncol. 2024 Jun 12;14:1377842. doi: 10.3389/fonc.2024.1377842 (PMC11199536; doi:10.3389/fonc.2024.1377842)
Supplement: Supplementary file 2 [file Table_1.docx]

Supplementary Material

# Supplementary Figures and Tables

| **Table S1. Summary of EV-related cutaneous events** |  |  |
| --- | --- | --- |
|  | Any grade  N=42 | Grade 3-4  N=7 |
| **Rash** | 32 (76.2) | 7 (100) |
| **Pruritus** | 23 (54.8) | 2 (28.6) |
| **Photosensitivity** | 5 (11.9) | 1 (14.3) |
| **Skin Hyperpigmentation** | 5 (11.9) | 1(14.3) |
| **Pain of skin** | 4 (9.5) | 1 (14.3) |
| **Bullous dermatitis** | 4 (9.5) | 1 (14.3) |
| **Rash Maculo-papular** | 2 (4.8) | 0 (0) |
| **Dry skin** | 2 (4.8) | 0 (0) |
| **Exfoliative rash** | 2 (4.8) | 1 (14.3) |
| **Skin ulceration** | 1 (2.4) | 0 (0) |
| **Rash acneiform** | 1 (2.4) | 0 (0) |

| **Table S2. Summary of dose modifications during treatment** | | |
| --- | --- | --- |
|  | **Cutaneous toxicity**  **N= 42** | **No cutaneous toxicity**  **N= 36** |
| **Patients who received dose reduction due to any EV-related toxicity**, n (%) ^1^ | 22 (52.4) | 5 (13.9) |
| Dose reduction due to cutaneous toxicity, n (%) | 12 (28.6) | 0(0) |
| Dose reduction due to peripheral neuropathy, n (%) | 6 (14.3) | 3 (8.3) |
| Dose reduction due to other EV-related toxicities, n (%)^2^ | 4 (9.5) | 2 (5.6) |
| **Patients who returned to full dose of EV after dose reduction,** n (%) **^3^** | 4 (9.5) | 2 (5.6) |
| **Patients who discontinued EV due to any EV-related toxicity,** n (%) | 9 (21.4) | 4 (11.1) |
| EV = enfortumab vedotin  ^1^ Reduced dose of EV was 1 m g/kg for all patients.  ^2^ Other EV-related toxicities included diarrhea, transaminitis, neutropenia.  ^3^ Full dose of EV is 1.25 mg/kg, capped at 125mg/dose | | |

| **Table S3. Multivariable Cox proportional hazard model analysis for PFS and OS with relevant baseline characteristics and EV-related peripheral neuropathy (n=78)** | | | | |
| --- | --- | --- | --- | --- |
| **Characteristics** | **PFS**  **HR (95% CI)** | **p-value** | **OS**  **HR (95% CI)** | **p-value** |
| **Peripheral Neuropathy** (time-dependent): Yes vs. No | 1.32 (0.72, 2.42) | 0.3747 | 1.04 (0.54, 1.99) | 0.9125 |
| **Gender**: Female vs Male | 1.08 (0.52, 2.25) | 0.8287 | 0.72 (0.32, 1.66) | 0.4466 |
| **Metastatic Disease**: Visceral metastases vs. Lymph nodes only | 1.66 (0.77, 3.55) | 0.1953 | 2.82 (0.92, 8.58) | 0.0686 |
| **ECOG PS**: |  |  |  |  |
| 1 vs. 0 | 1.5 (0.84, 2.68) | 0.1599 | 2.73 (1.32, 5.67) | 0.0053 |
| ≥2 vs. 0 | 2.1 (0.95, 4.64) |  | 4.17 (1.66, 10.5) |  |
| **Weight**, per one kilogram | 0.98 (0.96, 1.003) | 0.1004 | 0.98 (0.96, 1.01) | 0.1814 |
| **EV dose** (time dependent):  Reduced 1 mg/kg vs. Full 1.25mg/kg dose | 0.81 (0.48, 1.37) | 0.4312 | 0.58 (0.32, 1.07) | 0.0806 |
| ECOG PS: Eastern Cooperative Oncology Group performance status; EV: enfortumab vedotin | | | | |

**Supplementary Figure 1. Consort Flow diagram**

**
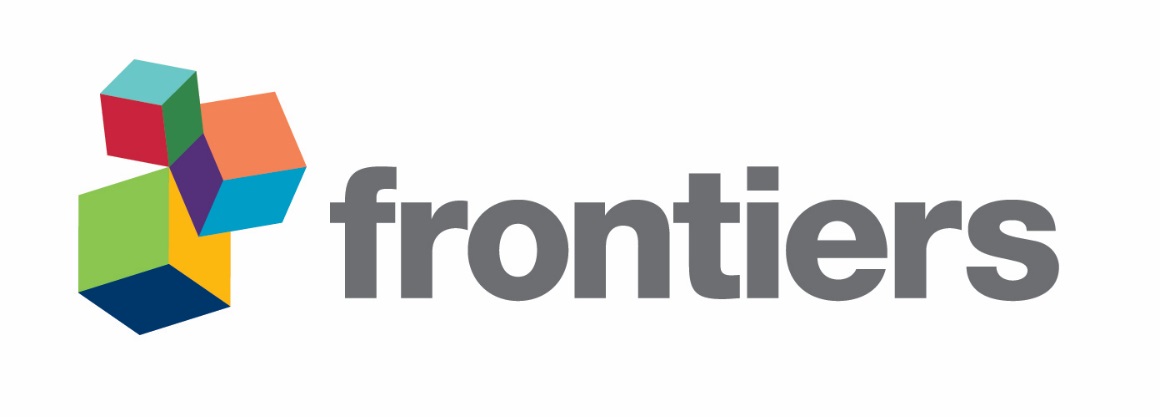
**
